# Effect of different timing of letrozole initiation on pregnancy outcome in polycystic ovary syndrome

**DOI:** 10.3389/fendo.2022.1059609

**Published:** 2022-11-24

**Authors:** Lan Shi, Shujin Ye, Mengyun Gao, Yijie Chen, Xuejing Jin, Zhifen Zhang

**Affiliations:** ^1^ Department of the Fourth Clinical Medical College, Zhejiang Chinese Medical University, Hangzhou, Zhejiang, China; ^2^ Department of Obstetrics and Gynecology, Hangzhou Women’s Hospital (Hangzhou Maternity and Child Health Care Hospital), Hangzhou, Zhejiang, China

**Keywords:** polycystic ovarian syndrome, letrozole, ovulation induction, infertility, conception, pregnancy

## Abstract

**Objective:**

To investigate the efficacy of oral letrozole (LE) starting on day 3 or 5 of the menstrual cycle in patients with polycystic ovary syndrome (PCOS).

**Design:**

Retrospective cohort study.

**Setting:**

Reproductive Endocrinology Department of Hangzhou Women’s Hospital.

**Methods:**

In this retrospective analysis, we analyzed patients who received oral LE for ovulation induction (OI) at the Hangzhou Women’s Hospital from January 2016 to January 2021. In total, 539 PCOS patients with fertility requirements were classified into the D3 group and D5 group according to the different starting times of oral LE, that is, from the 3rd or 5th day of the menstrual cycle or LE is taken orally for 5 days starting on day 3 or 5 of progesterone withdrawal bleeding. Treatment started with one tablet (LE 2.5 mg), continue the regimen from the previous cycle in non-responders and continued until pregnancy or for up to three ovulatory cycles, with visits to determine ovulation and pregnancy, followed by tracking of pregnancies. The primary outcome was to compare ovulation rates, conception rates, live birth rates, pregnancy complications, and pregnancy outcomes at different initiation times.

**Results:**

Women who started LE on the 5th day of their menstrual cycle had more cumulative conception rates than those who started LE on the 3rd day(173 of 228[75.9%]*vs*. 201 of 311[64.6%], P= 0.005; rate ratio for conception, 1.174; 95% confidence interval,1.052 to 1.311) without significant differences in overall live birth rate, though there were 142 of 228[62.3%] in the D5 group versus 172 of 311[55.3%] in the D3 group (P= 0.105). The median (IQR) endometrial thickness was significantly (P = 0.013) greater during the D5 group treatment compared to the D3 group, which may be related to higher conception and clinical pregnancy rates. The median (IQR) maximum follicle diameter was not statistically (P = 0.073) different between the two groups. The cumulative ovulation per cycle rate was higher with D5 than with D3 (287 of 405 treatment cycles [70.9%] *vs*. 388 of 640 treatment cycles [60.6%], P=0.001). There were no significant between-group differences in pregnancy loss (31 of 173 conceptions in the D5 group [17.9%] and 29 of 201 conceptions in the D3 group [14.4%]) or multiples pregnancy (8.2% and 10.5%, respectively). Rates of other adverse events during pregnancy were similar in the two treatment groups.

**Conclusion:**

As compared with D3 group, D5 group was associated with higher ovulation and conception rates, shorter time-to-pregnancy among infertile women with the PCOS.

## Introduction

Polycystic ovary syndrome (PCOS) is the most common endocrine disorder in women of reproductive age, affecting about 10% of women (1). PCOS is characterized by sparse ovulation, hyperandrogenism, and polycystic ovary morphology based on ultrasound evaluation. It is usually associated with metabolic syndromes such as insulin resistance, obesity, hyperlipidemia, and hypertension. It accounts for 90% of infertility in anovulatory women and is one of the important causes of infertility in women of childbearing age (1). Current research shows that although the natural conception rate for women with PCOS is low, the treatments and strategies currently being used in the clinic are highly effective in improving conception rates (2). Therefore, choosing simple but effective infertility treatment options for patients with PCOS is essential. Even though PCOS is a complex disorder of reproductive metabolism, the hypothalamic-pituitary axis remains the target of first-line ovulation treatment. Many treatment options aimed at achieving ovulation, pregnancy, and live birth have been used with varying success (eg. Aromatase inhibitors, clomiphene, metformin for patients with abnormal glucose metabolism, etc.) (3, 4).

Letrozole (LE), the third generation of an aromatase inhibitor, is a new type that stimulates ovulation drugs to inhibit androstenedione and the conversion of testosterone to estrogen in the ovary, decreasing estrogen levels. It acts on the hypothalamic-pituitary gland through positive feedback, promotes the secretion and release of FSH, and induces follicle development and mature discharge (5). Numerous randomized trials have found increased ovulation, pregnancy, and live birth rates in women with PCOS after LE ovulation promotion compared to clomiphene citrate ovulation (6–8). Currently, LE is started on the third to the fifth day of the menstrual cycle. The potential advantages of using LE during this period are its relatively short half-life (*45 h), accumulation of intraovarian androgens, and activation of estrogen receptors, which will enhance follicular sensitivity, resulting in rapid endometrial growth. Nevertheless, there is no consensus on the optimal start time (9). We designed a retrospective cohort study to compare effectiveness and safety when LE was started on the third or fifth day of menstruation, respectively, to explore the optimal timing of ovulation initiation.

## Materials and methods

### Study oversight

This retrospective cohort study was approved by the Ethics Review Committee of the Hangzhou Women’s Hospital. Written informed consent was waived due to the retrospective nature of the study.

### Participants

A total of 624 patients with PCOS who received LE ovulation-promoting treatment and visited the Reproductive Endocrinology Department of Hangzhou Women’s Hospital from January 2016 to January 2021 were collected.

The inclusion criteria for participants eligible for the use of ovulation-promoting drugs were: 1) Women of childbearing age between 20 and 40 years old who have not been pregnant without contraception for ≥1 year; 2) The PCOS was defined according to Rotterdam Consensus 2003 (Meet two of the three and exclude other causes of hyperandrogenism: Low ovulation/anovulation, clinical manifestations of high androgen (acne/hirsute) and/or biochemical manifestations (testosterone≥0.8ng/ml or free androgen index [FAI]≥5)[3], gynecological ultrasonography during the menstrual cycle or 3rd to 5th days after bleeding after progesterone withdrawal suggests polycystic changes in the ovary (small follicles with a diameter of 2 -9 mm, ≥12 small follicles, and/or ovarian volume >10 ml) (10); 3) The women and their partners agreed to have regular intercourse with the intention of conception during the study.

The exclusion criteria include: 1)Women with BMI > 30kg/m2; 2)Patients with tubal factor infertility; 3)Patients with uterine and reproductive tract malformation confirmed by gynecological ultrasound, HSG, laparoscopy, or hysteroscopy; 4) Patients with infertility due to abnormalities in the male partner’s semen (normal sperm concentration of 15 million per milliliter and a normal activity rate of >40%, WHO 2010.); 5)Women who were pregnant before this ovulation induction drug started; 6)Women who have received ovulation induction (OI) treatment within 6 months and gonadotropin-releasing hormone agonists (GnRHa) within three months; 7) Patients with diabetes mellitus, hypertension, endometrial hyperplasia/cancer, thyroid disease, and hyperprolactinemia that cannot effectively control by medication; 8)Patients with major systemic illnesses; 9)Patients with a history of LE allergy and contraindications.

### Study overview

The final 539 patients were included and divided into groups D3 (n=311) and D5 (n=228) according to the start of oral LE(Femara, Novartis Pharmaceuticals), that is, oral LE 2.5 mg/d for 5 days starting on days 3 and 5 of the menstrual cycle. All patients were tested for follicular growth by vaginal ultrasound from day 10 of the menstrual cycle.

HCG (human chorionic gonadotrophin) injection and corpus luteum support standard: When the largest diameter of the dominant follicle was ≥18mm or the urine LH was positive, an intramuscular injection of HCG5000-10000 IU was used to induce ovulation, and the patient was asked to have sex on the injection day or the next day; or intrauterine insemination was performed 24h or 36h after HCG injection. After that, vaginal ultrasound monitoring is done daily until the day of ovulation, or every 2-3 days until after the next menstrual period if ovulation has not occurred after 96 hours of HCG.

Urine HCG testing 10 days after ovulation to determine conception, the follow-up to 5-6 weeks after the last menstrual period, diagnosis of clinical pregnancy when a gestational sac is detected by ultrasound, and the obstetric records of those conceiving were reviewed for pregnancy outcomes. If no conception occurs, continue the regimen from the previous cycle, with no conception for 3 consecutive cycles considered a failure.

Criteria for interrupting the treatment cycle: At the risk of ovarian hyperstimulation: ≥3 dominant follicles, ovarian diameter ≥60mm, ascites, serum estradiol level ≥5500pmol-L-1.

### Outcomes

The primary outcome of the study was the conception(serum or urine HCG was positive)and live birth rates. Secondary outcomes included the rate of ovulation(serum progesterone level over 5ng/ml within one cycle), endometrial thickness(on the day of intramural injection of HCG), maximum follicular diameter(on the day of intramural injection of HCG), treatment cycles received until pregnancy, pregnancy loss(including biochemical, miscarriage, ectopic), pregnancy outcome, multiples pregnancy, pregnancy complications, mode of delivery and other adverse events.

### Blood examination

Days 2-5 of the menstrual cycle, after a period of 10 or more hours without food, blood samples were collected from all participants before breakfast. Anti-Mullerian hormone (AMH), Follicle-stimulating hormone (FSH), luteinizing hormone (LH), Progesterone(P), Testosterone (T), Prolactin (PRL) and thyroid stimulating hormone (TSH) were measured using the chemiluminescence method (Beckman Coulter UniCel Dxl-800). The Beckman Coulter AU5821 chemistry analyzer was used to measure fasting plasma glucose (FPG), fasting insulin (FINS), total bilirubin, total cholesterol (TC), triglycerides (TG), high-density lipoprotein (HDL), low-density lipoprotein (LDL), Apoa1, homocysteine (HCY), uric acid (UA), serum calcium, serum phosphorus, neutrophil to lymphocyte ratio (NLR), platelet (PLT), and C-reactive protein (CRP).

### Statistical analysis

Statistical analysis was performed using SPSS Statistics 26. Participants’ characteristics in the two allocated treatment sequences were compared using independent non-parametric tests. Generalized estimating equations were used for the analysis of the ovulation rate to account for the correlation of multiple ovulation cycles for each subject. The endometrial thickness and maximum follicle diameter were compared using independent non-parametric tests. Categorical data were compared using the chi-squared test or Fisher’s exact test. In addition, the rate ratio (RR) and the absolute difference (AD) (95% Confidence Interval) were estimated for conception and live birth rates. Kaplan–Meier curves were used for time-to-event analyses. Logistic regression models created odds ratios (ORs) with associated 95 percent confidence intervals (CIs), which were used to assess the relationship between characteristics associated with conception and live birth after controlling for potential confounders such as maternal age and BMI (body mass index), LH/FSH, AMH (anti-Müllerian hormone), T (testosterone), and TSH (thyroid stimulating hormone).

## Results

### Characteristics of the patients

**Figure 1:**
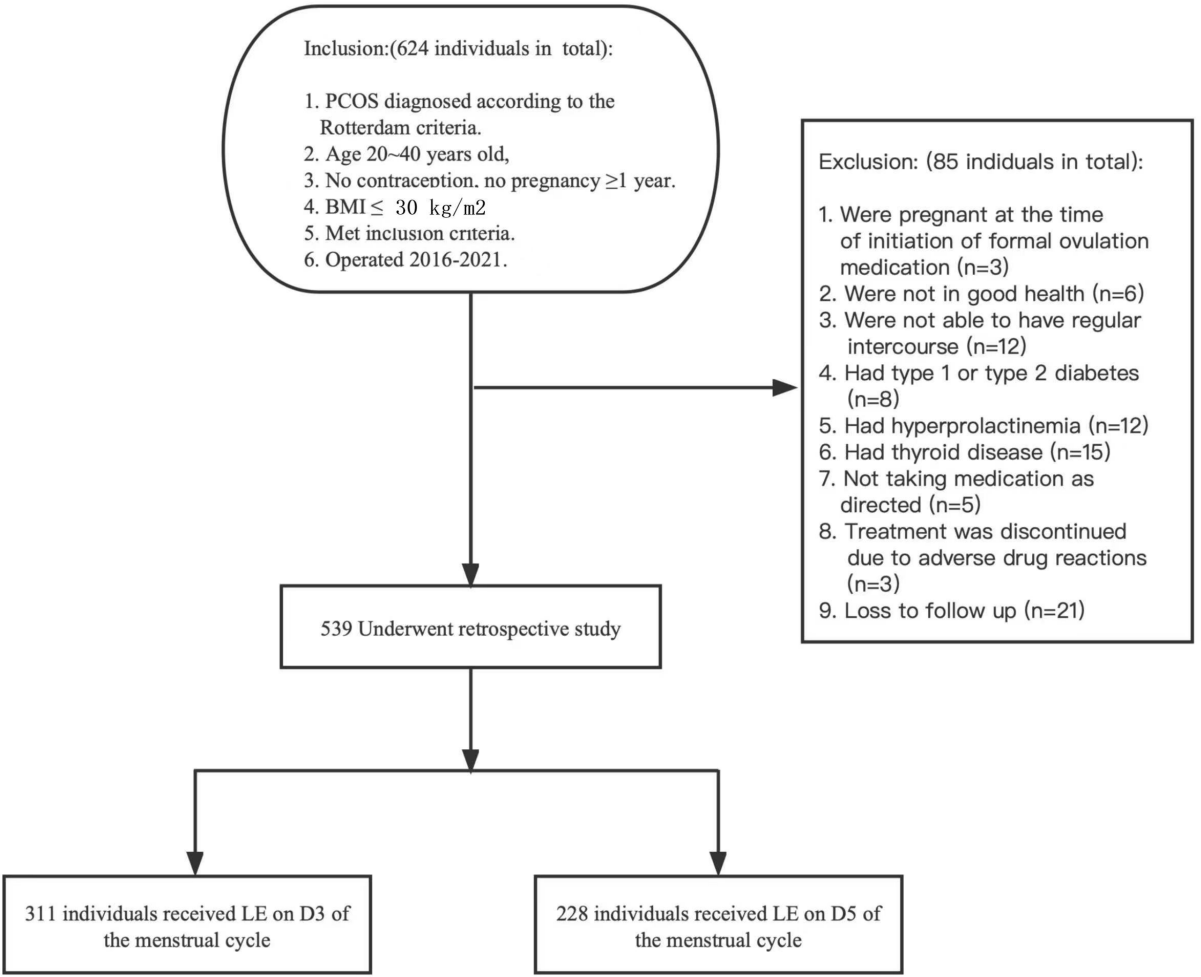
Flow Chart Depicting the Patient Selection.

**Figure 1** illustrates the flow of participants throughout the trial. In total,539 patients were included in this study, with 311 patients receiving LE on the 3rd day of their menstrual cycle and 228 patients receiving LE on the 5th day. Based on the baseline characteristics of both groups in **Table 1**, no significant differences were found at baseline characteristics.

**Table 1:** Baseline Characteristics of Participants.

	D3 (n = 311)	D5 (n = 228)	P
Age (years)	28.0 (26.0 to 31.0)	29.0 (26.0 to 31.0)	0.434
BMI (kg/m^2)	22.0 (19.8 to 24.3)	22.1 (19.8 to 24.1)	0.801
Infertility duration (years)	2.0 (2.0 to 2.0)	2.0 (2.0 to 3.0)	0.059
AMH (ng/ml)	6.5 (4.2 to 10.0)	7.0 (4.2to 10.0)	0.833
LH (IU/L)	7.4 (4.6 to 13.1)	7.8 (5.1 to 13.7)	0.39
FSH (IU/L)	6.7 (5.6 to 7.7)	6.4 (5.2 to 7.7)	0.208
P (ng/ml)	0.62 (0.37 to 1.02)	0.62 (0.43 to 1.08)	0.218
Testosterone (ng/ml)	0.57 (0.44 to 0.72)	0.61 (0.42 to 0.77)	0.064
FAI (%)	6.56 (3.8 to 11.0)	5.8 (2.9 to 11.2)	0.419
Prolactin (ng/ml)	11.9 (8.9 to 16.8)	12.2 (9.1 to 18.3)	0.414
Fasting insulin (mIU/L)	6.7 (4.6 to 10.6)	8.2 (5.7 to 10.7)	0.146
Fasting glucose (mmol/L)	5.0 (4.6 to 5.2)	4.9 (4.6 to 5.2)	0.605
TSH (mIU/L)	1.8 (1.4 to 2.6)	1.9 (1.5 to 2.6)	0.921
PLT (10^9/L)	245 (212 to 275)	237 (209 to 273)	0.313
NLR	1.8 (1.4 to 2.5)	2.0 (1.5 to 2.7)	0.126
UA (umol/L)	296 (253 to 347)	304 (255 to 354)	0.557
TC (mmol/L)	4.92 (4.32 to 5.54)	4.78 (4.27 to 5.52)	0.464
TG (mmol/L)	1.08 (0.74 to 1.72)	1.12 (0.82 to 1.66)	0.406
HDL (mmol/L)	1.52 (1.27 to 1.78)	1.50 (1.27 to 1.79)	0.79
LDL (mmol/L)	2.62 (2.21 to 3.04)	2.61 (2.16 to 3.04)	0.661
Apoa1 (g/L)	1.50 (1.29 to 1.71)	1.44 (1.28 to 1.74)	0.536

(Numerical data presented as median (25th to 75th percentile).

D3, using LE on the 3rd day of menstruation; D5, using LE on the 5th day of menstruation.

BMI, body mass index; AMH, anti-Müllerian hormone; LH, luteinizing hormone; FSH, follicle-stimulating hormone; P, progesterone; FAI, free androgen index; TSH, thyroid stimulating hormone; NLR, neutrophil to lymphocyte ratio; UA, uric acid; TC, total cholesterol; TG, triglyceride; HDL, high density lipoprotein; LDL, low density lipoprotein.

### Primary outcomes (conception and live birth)

**Figure 2:**
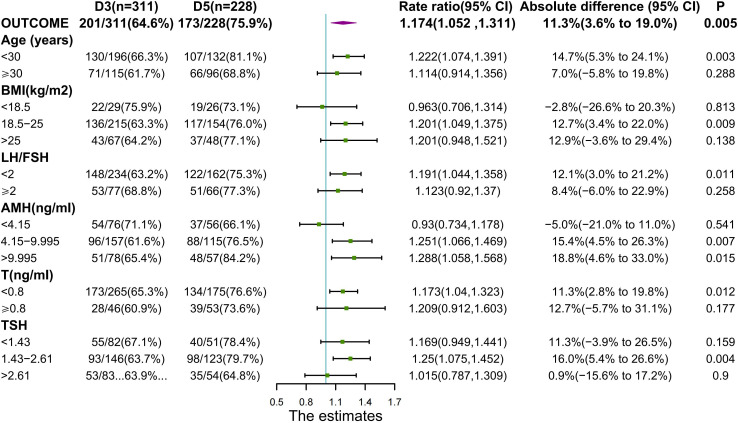
Outcomes with Regard to Conception Rate.

**Figure 3:**
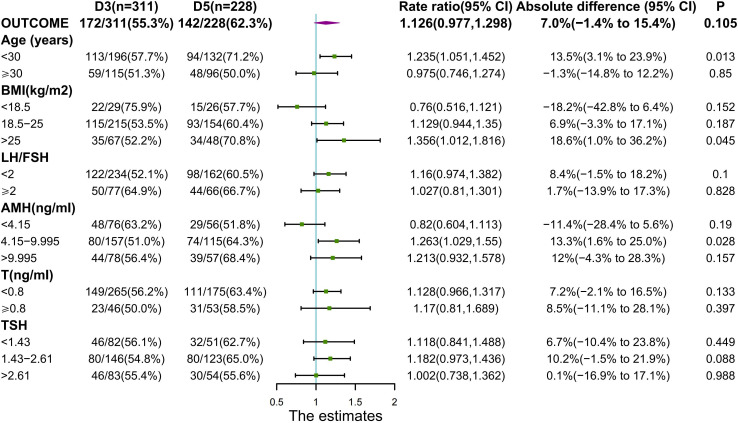
Outcomes with Regard to Live Birth Rate.

The conception and live birth rates were depicted in **Figure 2** and **Figure 3** for the overall and each stratum. Throughout the study, as compared with the D3 group, the D5 group exhibited a substantial increase in conception rates (173 of 228 women [75.9%] vs. 201 of 311 [64.6%], P=0.005; RR for conception on the 5th day, 1.174; 95% CI,1.052 to 1.311) (**Figure 2**).

We performed an analysis according to the maternal BMI, when BMI was 18.5-25(normal range), the D5 group had significantly greater conception rates than the D3 group (76.0% vs. 63.3%, p=0.009). In PCOS individuals with BMI >25, the D5 group had a significantly higher live birth rate than the D3 group (70.8% vs. 52.2%, p=0.045). There was no large discrepancy in conception or live birth rates between the two groups of PCOS patients with the BMI < 18.5.

Sub-analysis of conception rates based on different ages, LH/FSH, T, and TSH revealed between the two groups. When the age was <30, LH/FSH <2, AMH≥4.15, T<0.8, and TSH was 1.43-2.61, the D5 group had a greater conception rate than the D3 group, and all were statistically different.

There was no statistical difference in live birth rates between the two groups, although the 5th day arm had a trend toward higher rates. To detect variations in live birth rates, larger sample size is necessary. The primary outcome of live birth was significantly influenced by age (p=0.027) (**Table 2**), when the age was less than 30, the live birth rate for subjects in the D5 group was considerably greater than for women in the D3 group (p=0.013) (**Figure 3**). At AMH between 4.15 and 9.995, live birth rates were higher in the D5 group than in the D3 group (p=0.028) (**Figure 3**).

**Table 2:** Logistic Regression of Live Birth Outcome.

Baseline Parameter	Category	aOR (95% CI)	P
Age (years)	per year increased	0.944 (0.896 to 0.993)	0.027
BMI (kg/m2)	per kg/m2 increased	0.992 (0.942 to 1.046)	0.779
LH/FSH	per increased	1.214 (1.011 to 1.458)	0.037
AMH (ng/ml)	per ng/ml increased	1.011 (0.964 to 1.059)	0.66
T (ng/ml)	per ng/ml increased	0.775 (0.358 to 1.678)	0.519
TSH (mIU/L)	per mIU/L increased	0.929 (0.785 to 1.101)	0.396
Type of treatment	D3 vs. D5	1.325 (0.929 to 1.888)	0.12

Analyses were adjusted for maternal age, maternal BMI, LH/FSH, AMH, T, TSH.

aOR, adjusted odd ratio.

### Secondary outcomes (ovulation, pregnancy, and pregnancy loss)

On the day of HCG intramuscular injection, the median (IQR) endometrial thickness was substantially (P = 0.013) larger in the D5 group than in the D3 group. The maximum follicle diameter was not statistically (P = 0.073) different between the two groups (**Table 3**).

**Table 3:** Rates of Ovulation, Pregnancy, and Pregnancy Loss.

Variable	D3 (n = 311)	D5 (n = 228)	Rate ratio (95% CI)	Absolute difference (95% CI)	P
No. of ovulations/total treatment cycles	388/640 (60.6%)	287/405 (70.9%)		10.2% (4.3% to 15.9%)	0.001
ET (mm) [median (IQR)]	8.0 (7.0 to 10.0)	9.0 (8.0 to 10.0)			0.013
Maximum follicle diameter	1.88 (1.77 to 1.97)	1.84 (1.75 to 1.95)			0.073
Conception	201/311 (64.6%)	173/228 (75.9%)	1.174 (1.052 to 1.311)	11.3% (3.6% to 19.0%)	0.005
Pregnancy	172/311 (55.3%)	146/228 (64.0%)	1.158 (1.007 to 1.331)	8.7% (0.4% to 17.0%)	0.042
Singleton	154/172 (89.5%)	134/146 (91.8%)	1.025 (0.955 to 1.1)	2.3% (-4.1% to 8.7%)	0.495
Twins	16/172 (9.3%)	12/146 (8.2%)	0.884 (0.432 to 1.807)	-1.1% (-7.3% to 5.1%)	0.734
Multiples	18/172 (10.5%)	12/146 (8.2%)	0.785 (0.391 to 1.576)	-2.3% (-8.7% to 4.1%)	0.495
Pregnancy loss					
Total losses among subjects who conceived	29/201 (14.4%)	31/173 (17.9%)	1.242 (0.781 to 1.975)	3.5% (-4.0% to 11.0%)	0.359
Biochemical factor or no fetal heart motion	21/201 (10.4%)	20/173 (11.6%)	1.107 (0.621 to 1.972)	1.1% (-5.3% to 7.5%)	0.731
Ectopic pregnancy	8/201 (4.0%)	7/173 (4.0%)	1.017 (0.376 to 2.746)	0.1% (-4.0% to 4.0%)	0.974
Loss after observed heart motion	0/201 (0.0%)	4/173 (2.3%)		2.3%0.1% to 4.5%)	0.045
Events among ovulated cycles					
Conception	201/388 (51.8%)	173/287 (60.3%)	1.164 (1.017 to 1.331)	8.5% (0.9% to 15.9%)	0.029
Singleton pregnancy	154/388 (39.7%)	134/287 (46.7%)	1.176 (0.988 to 1.4)	7.0% (-0.5% to 14.5%)	0.069
Singleton live birth	154/388 (39.7%)	130/287 (45.3%)	1.141 (0.956 to 1.362)	5.6% (-2.0% to 13.1%)	0.145
Events among subjects who ovulated					
Conception	201/287 (70.0%)	173/210 (82.4%)	1.176 (1.066 to 1.298)	12.4% (4.7% to 19.5%)	0.002
Singleton pregnancy	154/287 (53.7%)	134/210 (63.8%)	1.189 (1.025 to 1.379)	10.2% (1.4% to 18.6%)	0.024
Singleton live birth	154/287 (53.7%)	130/210 (61.9%)	1.154 (0.992 to 1.342)	8.3% (-0.6% to 16.8%)	0.067

Categorical data: % (n/N); Ovulation was defined as serum progesterone level over 5ng/ml within one cycle; IQR, interquartile; ET, endometrial thickness (on the day of intramural injection of HCG); Conception was defined as a serum level of human chorionic gonadotropin that was positive; Pregnancy was defined by the presence of fetal heart movements on ultrasound.

Per cycle analysis revealed considerably greater ovulation rates in the D5 group (P=0.001) (**Table 3**). As previously stated, however, the variations in ovulation rates did not result in a significant increase in the live birth rate among the D5 group. Rates of conception and Singleton pregnancy per cycle in which ovulation occurred were higher in the D5 group (P=0.029, P=0.069). There was statistically significant change in the frequencies of conception and singleton pregnancy per ovulated subject (P=0.002, P=0.024). But there was no statistically significant change in the frequencies of live births per ovulated subject.

**Figure 4:**
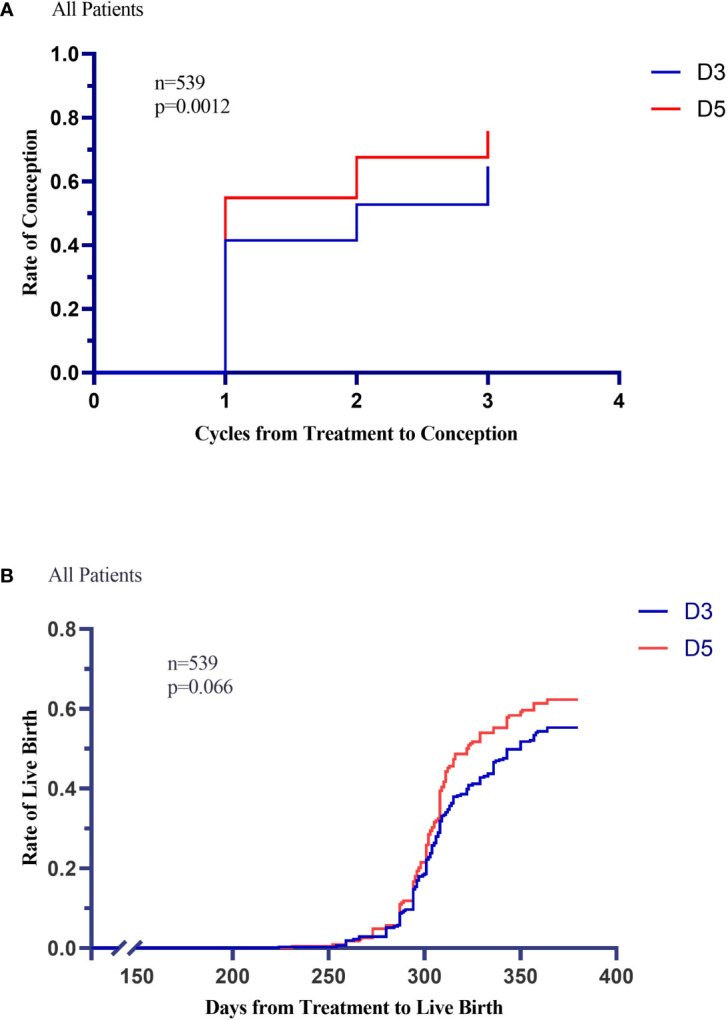
Kaplan-Meier Curves for Conception and Live Birth. Conception rates and Live-birth rates are shown according to treatment group in Panel **A** and Panel **B**.

The median number of treatment cycles received until pregnancy was significantly (log-rank P=0.0012) smaller with the D5 group (2[1–3] cycles) compared to the D3 group (1[1–3] cycles) (**Figure 4**).

The pregnancy rate in the D3 group was considerably lower than in the D5 group (P=0.042 for both comparisons). The rates of pregnancy loss after conception were equivalent in the two treatment groups. Four adverse events of pregnancy loss after observed heart motion occurred during infertility treatment in the D5 group (**Table 3**).

Between the D3 and D5 groups, there was no significant difference in the period from treatment initiation to live birth (log-rank P=0.66) (**Figure 4**).

### Adverse events and mode of delivery

**Table 4** summarizes adverse events and their mode of delivery. During pregnancy, gestational diabetes was the most prevalent consequence, followed by early membrane rupture, preterm labor, and HDP (hypertensive disorder in pregnancy), with no significant differences between treatment groups. There were two substantial congenital malformations (one with D3 and one with D5); the between-group difference was not significant. Both groups had similar delivery patterns.

**Table 4:** Adverse Events.

Event	D3 n (%)	D5 n (%)	Rate ratio (95% CI)	P
sPTB	21 (10.4%)	17 (9.8%)	0.941 (0.513 to 1.725)	0.843
Eutocia	95 (47.3%)	77 (44.5%)	0.942 (0.755 to 1.175)	0.594
Cesarean section	71 (35.3%)	63 (36.4%)	1.031 (0.786 to 1.353)	0.826
Forceps delivery	6 (3.0%)	2 (1.2%)	0.387 (0.079 to 1.894)	0.295
GDM	40 (19.9%)	29 (16.8%)	0.842 (0.546 to 1.298)	0.435
HDP	16 (8.0%)	15 (8.7%)	1.089 (0.555 to 2.138)	0.804
PROM	34 (16.9%)	32 (18.5%)	1.094 (0.706 to 1.694)	0.689
Fetal anomalies	1 (0.5%)	1 (0.6%)	1.162 (0.073 to 18.437)	1.0
LBW	7 (3.5%)	7 (4.0%)	1.162 (0.416 to 3.247)	0.775
VLBW	2 (1.0%)	2 (1.2%)	1.162 (0.165 to 8.161)	1.0
Macrosomia	5 (2.5%)	4 (2.3%)	0.929 (0.254 to 3.407)	1.0

sPTB, spontaneous preterm birth; GDM, gestational diabetes; HDP, hypertensive disorder in pregnancy; PROM, premature rupture of membranes; LBW, Infant low birth weight; VLBW, Infant very low birth.

### Logistic regression of conception and live birth outcome

**Table 5** shows the outcomes of a logistic regression model for the factors that could have influenced the conception rate. The results showed that using LE on the fifth day of menstruation had a substantial positive (favorable) effect (OR = 1.71, 95% CI: 1.161–2.519).

**Table 5:** Logistic Regression of Conception Outcome.

Baseline Parameter	Category	aOR (95% CI)	P
Age (years)	per year increased	0.96 (0.909 to 1.014)	0.146
BMI (kg/m2)	per kg/m2 increased	0.982 (0.929 to 1.039)	0.538
LH/FSH	per increased	1.184 (0.968 to 1.45)	0.1
AMH (ng/ml)	per ng/ml increased	1.03 (0.979 to 1.085)	0.256
T (ng/ml)	per ng/ml increased	0.743 (0.323 to 1.707)	0.484
TSH (mIU/L)	per mIU/L increased	0.856 (0.716 to 1.023)	0.087
Type of treatment	D3 *vs*. D5	1.71 (1.161 to 2.519)	0.007

Analyses were adjusted for maternal age, maternal BMI, LH/FSH, AMH, T, TSH.

aOR, adjusted odd ratio.

However, there was no significant effect of LE use on 5th day or 3rd day and a favorable effect of LH/FSH on live birth (OR = 1.214, 95 percent CI: 1.011-1.458) in regression models of live birth rates. Maternal age showed a negative (adverse) effect on outcome (OR=0.944, 95% CI: 0.896-0.993) (**Table 2**).

## Discussion

To the best of our knowledge, this is the first sufficiently powered clinical study exploring the optimal initiation time of LE. Our study found that starting LE on the fifth day of their menstrual cycle was more effective as a fertility treatment than starting LE on the third day in anovulatory women with PCOS. Ovulation, endometrial thickness (on the day of intramural injection of HCG), conception, and pregnancy were significantly more likely after treatment with the D5 group. The rate of pregnancy loss, mode of delivery, multiples pregnancy, and rates of other adverse events during pregnancy (including anomalies) did not differ significantly between the two treatment groups. Our study also found that the D5 group required 1.39 cycles on average to conceive, while the D3 group required 1.54 cycles on average. When studying fertility treatment, we acknowledge that the ideal primary outcome would be live birth. The live birth rate was 10% higher in the D5 group than in the D3 group (RR = 1.126), but this did not reach statistical significance because the study was not strong enough to look at this outcome (power = 37.4%, alpha = 0.05), so we cannot rule out possible differences with small sample sizes. At the same time, we consider that the outcome of live birth is related to many other factors during pregnancy, such as environmental, physical, dietary, exercise and lifestyle factors. Our research was a retrospective analysis, and all pregnant women were not managed uniformly and followed up regularly during pregnancy, so we chose the conception and ovulation rates as the primary outcome, which is a limitation of our study. We will expand the sample size and provide appropriate management for prospective randomized clinical trials. Regular follow-up of all pregnant women included in the trial criteria to exclude other factors that interfere with pregnancy outcomes where possible and further explore the effect of different LE initiation times on pregnancy outcomes.

The triad that determines the success of human reproduction is the embryo, the endometrium, and their interactions. Past high-quality studies have shown that women with PCOS exhibit a clinically significant increased risk of obstetric and neonatal complications (11). Similar results have been published previously, confirming that endometrial dysfunction and altered oocyte capacity in women with PCOS are well-documented mechanisms contributing to increased pregnancy complications and outcomes (12, 13). Women with PCOS can exert a potentially adverse effect on the endometrium with pre-conception, conception, and post-conception endometrial function. LE is a third-generation, highly effective, and specific aromatase inhibitor that does not bind to estrogen receptors and does not affect the quality of cervical mucus or the thickness, morphology, and tolerability of the endometrium. Past studies have demonstrated the ability of LE to improve endometrial development, and it is more conducive to embryo implantation (14). During controlled ovarian stimulation (COS), LE administration during the early follicular phase significantly increases testosterone and androstenedione levels in the follicular fluid, thereby improving the follicular sensitivity to FSH stimulation and optimizing pregnancy outcomes. Our data indicate that multiples pregnancy and rates of other adverse events during pregnancy (including anomalies) are similar in the two treatments we evaluated. The rate of multiple pregnancies is consistent with a randomized, double-blind study (6.1%) (6). Our Pregnancy loss with LE on D3 and D5 was similar (p=0.359), and this data was in general agreement with the results of a study that included 42 RCTs in which Pregnancy loss was 19% (7). Our data show that using LE on the fifth day of menstruation does not increase the risk of teratogenicity or miscarriage compared to using LE on the third day.

There have been conflicting reports about the timing of LE’s medication. Kaitlin et al. found that a single oral dose of LE 25 mg for 1 day versus 5 mg/d for 5 days resulted in comparable cycle pregnancy rates in both groups (14.2% vs. 11.6%) (15); Mitwally et al. showed that a single oral dose of LE 20 mg on day 3 of the menstrual cycle compared with 2. 5 mg/d on days 3-7 of the menstrual cycle resulted in comparable cycle pregnancy rates in both groups (15% vs. 18%) (16); Badawy et al. discovered that oral administration of LE 2.5mg/d on days 1-10 of the menstrual cycle resulted in a higher pregnancy rate than patients who received 5mg/d on days 1-5 of the menstrual cycle (17. 4% vs.12. 4%) (17); Other scholars believe that if there is no dominant follicle in the ovary and endometrial lesions are ruled out, any time during the follicular phase can be used as the time to initiate ovulation-promoting drugs. Nonetheless, the most widespread clinical use is for 5 days starting on day 3 or 5 of the menstrual cycle or progesterone withdrawal bleeding. Because LE is taken orally from day 3 or 5 of the menstrual cycle, it can pass a half-life (45 hours), which is right in the selection period of dominant follicles (5-7 days of the menstrual cycle) (18). Therefore, this method of medication has long been considered the clinic’s most reasonable ovulation induction plan.

However, no studies have compared the effect of oral LE starting on the third day and fifth day of the menstrual cycle. In our study, LE initiation on the 5th day of the menstrual cycle had higher ovulation rates, endometrial thickness (on the day of HCG intramuscular injection), pregnancy rates, and clinical pregnancy rates in women with PCOS than LE initiation on the 3rd day of the menstrual cycle. In the present study, though the median (IQR) maximum follicle diameter in the D3 and D5 groups were comparable on the day of intramuscular injection of HCG(P=0.0730), the median (IQR) endometrial thickness was significantly better in D5 group [9.0(8.0 to 10.0)mm] compared with D3 group [8.0(7.0 to 10.0)mm]. Roy et al. reported similar results in a randomized control trial of LE versus CC in women undergoing superovulation, which showed that vascular penetration of the endometrium was associated with thicker endometrium (19). At the same time, Chien et al. confirmed a significant increase in pregnancy rate with deeper endometrial vascular penetration (20). Therefore, we believe that the endometrium on the day of ovulation when LE started on the fifth day of menstruation is thicker than when LE started on the third day of menstruation. For the D5 group, the thickness of the endometrium is more compatible with the dominant follicles, and the permeability of the endometrial vascular is more profound. HCG was routinely administered to allow follicle maturation and precisely the time the intercourse for these couples to increase the conception rate of patients with PCOS favorably.

LE has a high cumulative ovulation rate in the trial by Legro et al.(61.7%), comparable to the cumulative ovulation rate in our D3 group (60.6%). However, the study was biased towards obese subjects, which may account for the lower live birth rate (8). The D5 group resulted in an increased pregnancy rate compared to the D3 group (RR 1.158, 95% CI 1.007 to 1.331). Our pregnancy rate was higher than that of the most recent Cochrane review (35%) (21), and we had obese patients lose weight before our OI treatment may have contributed to this. Moreover, our small sample size could be a contributing factor. However, a high-quality study found that PCOS patients using LE had a pregnancy rate of 61.2%, which is comparable to our findings (6).

A possible explanation for the higher success of using LE on D5 is the greater ovulation rate per cycle in the D5 group. Aromatase activity occurs in granulosa cells of follicles larger than 6-8 mm on Day 5-8 of the menstrual cycle. The dominant follicle produces more estradiol-17 than the cohort’s other follicles, inhibiting FSH. LE administered on day 5 of menstruation effectively inhibits aromatase activity, enlarges the FSH window to stimulate follicle growth, and enables the simultaneous selection of multiple follicles. However, past researches indicates that around one-fourth of apparently healthy women may experience more than one follicle selection during a single menstrual cycle. It is conceivable that the variability of follicular dynamics in women of reproductive age is more significant than previously believed. We speculate that more than one follicular selection was probably generated in the D5 group (22).

Many studies have pointed to a decreasing trend in pregnancy rates as the age of the mother increases (23). In our research, we found that the conception rate was significantly higher in the D5 group than in the D3 group when the age was <30 years (81.1% vs. 66.3%, P=0.003), and the live birth rate was better in the D5 group than in the D3 group (71.2% vs. 57.7%, P=0.013). Therefore, for PCOS patients of optimal reproductive age, initiating LE on the 5th day of the menstrual cycle is the best time to promote ovulation.

BMI is one of the most important factors affecting fertility and pregnancy outcomes in women of childbearing age, obesity is responsible for an increased risk of subfecundity and infertility, and obesity has a significant impact on different PCOS phenotypes (24). For PCOS patients in different BMI groups, our study found that among PCOS patients whose BMI was 18.5-25 (normal weight), the pregnancy rate in D5 group was higher than that in D3 group (76.0% *vs*.63.3%, P=0.009). Our PCOS participants met the generally accepted Rotterdam diagnostic criteria for PCOS and had a median BMI of about 22 kg/m2. We believe that this cohort is well representative of PCOS women who receive fertility treatment at most fertility centers. Therefore, our results can be generalized to clinical practice on a global scale.

Past studies have shown that in the pathogenesis of PCOS, the secretion pattern of gonadotropin-releasing hormone (GnRH) is disrupted, leading to a relative increase in the release of LH and FSH. Although the LH/FSH ratio is not part of the androgen excess society’s diagnostic criteria for PCOS, in healthy women, the ratio of LH/FSH is usually between 1 and 2. In women with PCOS, this ratio flips. In some cases, it can reach 2 or 3. Ovulation does not occur in patients with PCOS due to the high LH/FSH ratio (25). Because the LH/FSH cut-off point is thought to indicate the responsiveness of ovaries to ovulation-stimulating medications, studies on the link between the LH/FSH cut-off point and pregnancy outcomes have been done since 1995 (26). Su et al. found that baseline LH/FSH level was a significant independent risk factor for live birth (p<0.05) (27). Our study found that when LH/FSH<2, the D5 group had a higher conception rate than the D3 group, with significant statistical differences. After adjusting for confounders, each unit increase in LH/FSH resulted in a 21.4% increase in a live birth. The small sample size could be the reason for this difference.

AMH is a product of the granulosa cells of the antral follicles, and serum AMH levels are 2-5 times higher in women with PCOS than in normal subjects due to the increased number of small follicles and excessive production of AMH per follicle (27, 28). We performed an analysis according to the quartile of maternal AMH. When AMH is in the 25th to 100th percentile range, the pregnancy rate of the D5 group is significantly higher than that of the D3 group. When AMH is in the 25th to 75th percentile range, The live birth rate of the D5 group was better than the D3 group, and the difference was statistically significant. Therefore, it can be inferred that the initiation of LE ovulation induction therapy on the fifth day of the menstrual cycle is more suitable for most anovulation PCOS patients.

Hyperandrogenemia is a prominent feature of PCOS and plays a significant role in its pathogenesis (29). Studies have shown that hyperandrogenemia in PCOS may alter the growth and function of early-onset follicles, leading to abnormal follicular development, which may affect conception and pregnancy (30). Our study found that among patients with T <0.8ng/mL, the pregnancy rate in the D5 group was significantly higher than that in the D3 group (76.6% vs. 65.3%, P=0.012). When T≥0.8ng/mL, the pregnancy rate in the D5 group was higher than that in the D3 group, but the difference was not statistically significant (P=0.177). Considering the sample size, we cannot rule out potential differences, which need to be confirmed by further studies.

Furthermore, we discovered that conception rates were significantly higher in the D5 group when TSH was in the 25th-75th percentile range. Logistic regression studies revealed that TSH levels were not substantially related to conception outcomes. Some studies have reported that TSH levels of 2.5 mIU/L did not link with fecundity, pregnancy loss, or live birth (31). That is similar to our findings, where higher D5 group conception rates contributed to identifying novel treatment options in the long term. The increase in E2 (estradiol) levels reduces free thyroid-hormone levels and increases the release of thyrotropin-releasing hormone (TRH), causing TSH levels to rise as a result of ovarian stimulation (32). That might explain the difference in conception rates between the two groups with high TSH levels.

Our retrospective study had several following limitations. First of all, the sample size was still relatively small, which may affect the results of this study. Then, this study did not manage patients uniformly during the pregnancy because it only analyzed the outcome data based on the completed cases, these intrinsic limitations, may impact its results. Third, this study did not utilize randomization and blinding, which may increase the risk of case selection. Finally, the 2018 guidelines proposed multiple phenotypes of PCOS (33), and our study did not further explore the optimal timing of ovulation induction for LE initiation in PCOS patients with different phenotypes.

In summary, our data showed that women who started LE on the fifth day of their menstrual cycle were superior to those who started LE on the third day as a treatment for anovulatory infertility in women with PCOS. The D5 group was associated with higher ovulation and conception rates and shorter time-to-pregnancy. These novel results suggest that this simple strategy may be an alternate low-risk, low-cost infertility treatment that offers superior reproductive results. Therefore, we recommend initiating LE on the fifth day of the menstrual cycle may be the best time for OI therapy with PCOS women. A further larger size of prospective randomized controlled studies is needed to clarify the optimal start time of LE OI therapy.

## Data availability statement

The original contributions presented in the study are included in the article/supplementary material. Further inquiries can be directed to the corresponding author.

## Ethics statement

The studies involving human participants were reviewed and approved by The Ethics Review Committee of the Hangzhou Women’s Hospital. Written informed consent for participation was not required for this study in accordance with the national legislation and the institutional requirements.

## Author contributions

ZZ and LS contributed to the conception of study. LS and SY were responsible for study designing, statistical analyses, and manuscript writing. ZZ and XJ contributed to revising the manuscript. LS, MG, and YC contributed to collecting data. All authors contributed to the article and approved the submitted version. LS and SY have contributed equally to this work.

## Funding

This study was supported by grants from the Zhejiang Province Major Science and Technology Program of Medicine and Health [No.WKJ-ZJ-2010] and Hangzhou City Major Science and Technology Program of Medicine and Health [Z20200007].

## Acknowledgments

We would like to thank the medical staff and patients in the Hangzhou Women’s Hospital for recording the data and cooperating with the treatment.

## Conflict of interest

The authors declare that the research was conducted in the absence of any commercial or financial relationships that could be construed as a potential conflict of interest.

## Publisher’s note

All claims expressed in this article are solely those of the authors and do not necessarily represent those of their affiliated organizations, or those of the publisher, the editors and the reviewers. Any product that may be evaluated in this article, or claim that may be made by its manufacturer, is not guaranteed or endorsed by the publisher.
